# Arrhythmogenic Right Ventricular Dysplasia/Cardiomyopathy Type 1: A Light on Molecular Mechanisms

**DOI:** 10.1155/2013/460805

**Published:** 2013-12-12

**Authors:** Koen L. A. Vanderschuren, Tom Sieverink, Ronald Wilders

**Affiliations:** Heart Failure Research Center, Academic Medical Center, University of Amsterdam, Meibergdreef 15, P.O. Box 22700, 1100 DE Amsterdam, The Netherlands

## Abstract

Arrhythmogenic right ventricular dysplasia/cardiomyopathy (ARVD/C) is an inherited cardiomyopathy associated with cardiac arrhythmias originating in the right ventricle, heart failure, and sudden cardiac death. Development of ARVD/C type 1 has been attributed to differential expression of transforming growth factor beta 3 (TGF**β**3). Several mechanisms underlying the molecular basis of ARVD/C type 1 have been proposed. Evaluating previously described mechanisms might elucidate how TGF**β**3 contributes to disease progression in ARVD/C type 1. Here we review how TGF**β**3 can induce fibrogenesis through Smad and/or **β**-catenin signaling. Moreover, the role of apoptosis is addressed. Finally the extent to which the immune system has been demonstrated to be a modulating and amplifying agent in the onset and progression of ARVD/C in general is discussed.

## 1. Introduction

Arrhythmogenic right ventricular dysplasia/cardiomyopathy (ARVD/C), also known as ARVD, ARVC, and ARVC/D, is an inherited cardiomyopathy. It is associated with cardiac arrhythmias originating in the right ventricle, heart failure, and sudden cardiac death [1]. Demanding physical activity is a significant risk factor for the development of ARVD/C. The estimated prevalence of ARVD/C varies between 1 in 2,000 and 1 in 5,000 [2, 3], but a higher prevalence has also been suggested [4, 5]. A higher prevalence of ARVD/C in male is a consistent finding, with an approximate ratio of 3 : 1 according to review papers [1, 2]. The actual ratio is, however, highly variable [6–11]. Most forms of ARVD/C are inherited in an autosomal dominant manner. Only two forms, that is, Naxos disease and Carvajal syndrome [12], are autosomal recessive diseases. Even though most forms of ARVD/C are autosomal dominant, disease penetrance is often incomplete and variable expression is observed, even among members of the same family [13]. This variability is poorly understood, and it significantly complicates genetic counseling [14].

**Figure 1:**
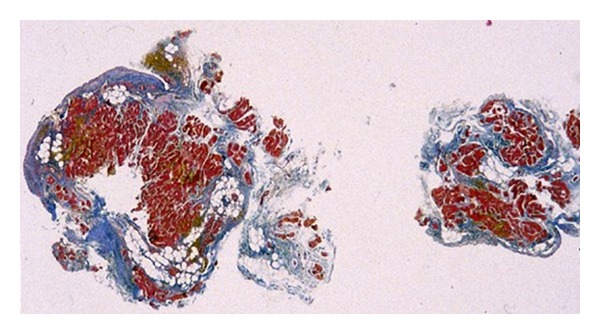
Two myocardial biopsies from the right ventricle of an ARVD/C patient. Heidenhain trichrome (red for myocytes, blue for fibrous tissue, and white for fatty tissue). Signs of fibrofatty infiltration are clearly visible. From Thiene et al. [18].

To help establish a diagnosis, Task Force Criteria have been proposed by McKenna et al. [15] and revised by Marcus et al. [16] to assure diagnostic sensitivity and specificity. Not all criteria must be fulfilled to recognize the disease as ARVD/C. A first criterion is global and/or regional structural alterations and cardiac dysfunction, which is manifested as a reduction of the right ventricular ejection fraction. The disintegration of the cardiac wall is characterized by gradual fibrofatty replacement in the myocardium [17, 18], as illustrated in Figure 1. The progressive thinning of the ventricular wall can eventually lead to aneurysms in the right ventricular wall. Moreover, repolarization abnormalities, such as inverted T-waves in the right precordial leads (*V*_1_–*V*_3_), are major criteria. In addition, depolarization or conduction anomalies can be observed, for example, epsilon waves and late potentials, as well as arrhythmias, such as ventricular tachycardia [16]. Fibrofatty replacement can interfere with optimal conduction in the remaining cardiac tissue, thereby explaining the conduction abnormalities that are considered to be signs of ARVD/C [16].

As part of the Task Force Criteria, genetics may be a helpful tool in establishing or confirming a diagnosis. Several mutations associated with ARVD/C have been described to date. Five proteins, out of a total of twelve that are related to ARVD/C, are part of the desmosomal complex [19], and thus play a role in cell-cell adhesion. About 50–60% of patients with ARVD/C are estimated to have a mutation in genes associated with cardiac desmosomes [1, 14, 20–22]. The desmosomal proteins involved are desmoplakin (encoded by the *DSP* gene), plakophilin 2 (*PKP2*), desmoglein-2 (*DSG2*), desmocollin-2 (*DSC-2*), and junctional plakoglobin (*JUP*). The nondesmosomal proteins related to ARVD/C are desmin (*DES*), transmembrane protein 43 (*TMEM43*), transforming growth factor *β*-3 (*TGF*β*3*), lamin-A/C (*LMNA*), titin (*TTN*), phospholamban (*PLN*), and *α*-T-catenin (*CTNNA3*). Even though these genes are associated with the different types of ARVD/C, often the underlying mechanisms resulting in the disease phenotype remain uncertain.

Over the years, it has become clear that ARVD/C is not a structural defect present at birth, explaining the original term dysplasia, and that the disease can also be biventricular or even left dominant [3]. The aforementioned cardiac arrhythmias tend to occur in the early, “concealed” phase of the disease, in the absence of the extensive structural damage that characterizes the later phases, and sudden cardiac death is often the first clinical manifestation of the disease [23]. The cardiac arrhythmias are probably related to remodeling of gap junctions and downregulation of the sodium current, which may occur because desmosomes, gap junctions, and the voltage-gated sodium channels form a “triad” of molecules that actually interact with each other [24].

The present review will focus on potential mechanisms involved in the development of ARVD/C type 1 (OMIM: 107970). ARVD/C type 1 is a rare form of ARVD/C [13], found only in a few families. This type of ARVD/C fulfills the Task Force Criteria and is believed to result from differential expression of TGF*β*3. The genetic origin of the disease was first mapped in 1994 [25], but the causative mutants were not identified until 2005 [26]. For two families, the mutations associated with ARVD/C were positioned in 3′ UTR or 5′ UTR regions flanking the TGF*β*3 gene [26]. An earlier study failed to show mutations in the exons of TGF*β*3 [27]. As the UTR regions are not translated, ARVD/C type 1 is not the result of a misfolded protein, but rather differential expression of the gene. Beffagna et al. [26] demonstrated that the mutations in both 3′- and 5′-UTR yielded approximately a 2.5-fold increase in translation in a murine myoblast cell line. Whether the aforementioned mutations result in equally substantial changes in TGF*β*3 expression *in vivo* has not been verified. Nevertheless, it seems reasonable to presume that TGF*β*3 overexpression causes ARVD/C type 1.

In the more prominent forms of ARVD/C, resulting from mutations in desmosomal proteins, occasionally multiple mutations were observed in a single patient [28, 29]. Presence of multiple nucleotide variations was associated with increased disease penetrance [30]. This underscores the importance of genetic background for disease progression. However, the data available for ARVD/C type 1 are too limited to provide clues for the involvement of other mutations. Therefore, we will only discuss the role of TGF*β*3.

TGF*β*3 (GeneID: 7043) is a secreted protein involved in tissue development and differentiation as well as fibrogenesis [31]. However, the exact mechanisms underlying the symptoms observed in ARVD/C type 1 have not been established. The involvement of several mechanisms in fibrofatty infiltration and the development of electrical anomalies have been postulated for ARVD/C or ARVD/C type 1 specifically. Here, we provide an overview of mechanisms that are possibly involved in ARCD/C type 1. The presumed role of TGF*β*3 overexpression in fibrogenesis, cell-cell adhesion abnormalities, apoptosis, and the role of inflammatory injury in general in ARVD/C will be discussed.

## 2. Role of Increased TGF*β*3 Synthesis in Fibrogenesis

The extent of fibrosis in the heart is correlated with the severity of conduction anomalies, such as delayed activation [32]. However, prolonged QRS complexes, which result from regional differences in depolarization times, are not proportional to the degree of fibrofatty infiltration [33]. This suggests that other processes are involved as well in disease progression. Whether TGF*β*3 is directly involved in promoting fibrofatty infiltration in the myocardium, or that the replacement of myocardial cells is an indirect result of other processes, such as apoptosis, remains to be established.

**Figure 2:**
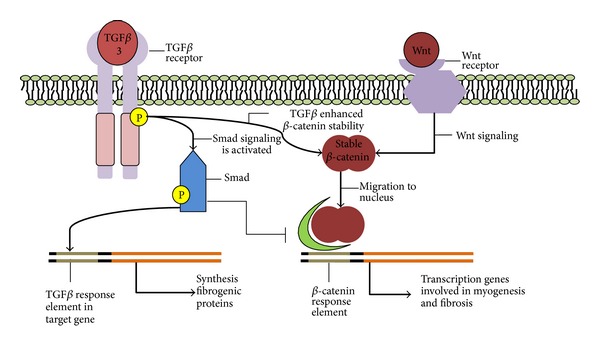
Proposed molecular mechanism underlying TGF*β*3-induced myocardial fibrofatty infiltration. Smad signaling is activated by TGF*β* receptors and induces fibrogenesis. Simultaneously, TGF*β* signaling enhances Wnt/*β* catenin signaling, whereas Smad proteins inhibit *β*-catenin altered gene expression. This augments fibrosis or hinders myogenesis, respectively. The net result is increased fibrofatty infiltration in cardiac tissue.

TGF*β*3 is known to be involved in tissue regeneration, accelerating healing and improving tissue strength, and it has been used to reduce scar tissue formation in wound healing [34]. This was achieved by better controlled deposition of extracellular matrix by fibroblasts, including collagen [34], implying a role for TGF*β*3 in extracellular matrix organization. Active TGF*β* is thought to affect fibroblasts through TGF*β* receptor 1 and 2 (TGFBR1 and 2), activating the Smad signaling pathway, as reviewed by Leask [35], and Shi and Massagué [36]. In this signaling pathway Smad proteins are phosphorylated by the TGF*β* receptor complex, and Smad 2 and 3 subsequently relay the signal to the nucleus, altering gene expression, as illustrated in Figure 2. It was shown that transforming growth factor beta 1 (TGF*β*1) overexpression induces cardiac fibrosis, resulting in an increased collagen content in the heart [37]. As TGF*β*3 exerts its function through the same signaling pathway, it seems possible that TGF*β*3 overexpression will also induce fibrosis in the myocardium. This is further corroborated by the presence of both TGF*β* 1 and 3 during cardiac fibrogenesis [38]. Moreover, decrease of Smad 3 levels was shown to attenuate fibrosis, by reduction in fibroblast activity [39, 40]. Thus, TGF*β* overexpression might excessively activate cardiac fibroblasts, resulting in fibrosis.

However, Smad activation *per se* might be insufficient to lead to ARVD/C. Persistent fibrosis resulting from TGF*β* overexpression requires cofactors [41], which might be an explanation for the lack of excessive fibrofatty replacement in other tissues of ARVD/C patients. Possibly the ventricular wall provides the right context for sustained fibrosis.

An alternative explanation for excessive fibrofatty tissue formation in the myocardium is episodes of apoptosis/necrosis. This would provide cofactors required for persistent fibrosis, as corroborated by several works, in which expression of these factors was observed after heart failure, dilated cardiomyopathy, and myocardial infarction [42, 43]. Moreover, a link has been suggested between inflammatory cytokines and an inflammatory cardiomyopathy resulting in cardiac failure and expression of cofactors required for sustained fibrosis [44].

Thus, it is likely that the myocardial fibrosis is the result of a complex interplay of intracellular signaling pathways, among which TGF*β*3-activated Smad signaling, on one side and the biochemical environment created in the tissue to sustain fibrosis on the other side. Disturbing this equilibrium by excessive TGF*β*3 expression may result in the augmented levels of fibrosis seen in ARVD/C.

## 3. Cell-Cell Adhesion Remodeling in ARVD/C Type 1

Clear signs of gap junction remodeling have been observed in ARVD/C patients [45–47], which might contribute to conduction and depolarization anomalies. Moreover, it has been hypothesized that the alterations in cell-cell adhesion in ARVD/C makes cardiac tissue vulnerable to exposure to physical stress [48]. As *β*-catenin is an integral part of the adherens junctions, loss of cell-cell adhesion inhibits Wnt/*β*-catenin signaling. This perturbed cell signaling pathway can result in induced cardiac myocyte apoptosis and development of fibrofatty tissue [19, 45]. Yet, exact mechanisms by which the interplay in cell-cell adhesion is altered remain uncertain [48]. Furthermore, no mechanisms have been proposed to explain similar effects induced by mutations in nondesmosomal proteins, although it is becoming increasingly clear that ARVD/C is a disease of the entire intercalated disc, in which there is a tight interaction between desmosomal and nondesmosomal molecules [3, 24].

It is unclear whether cell-cell adhesion remodeling is observed in ARVD/C type 1 as well, due to the limited number of individuals suffering from this disease. However, the disease phenotype of ARVD/C type 1 is very similar to other types of ARVD/C caused by mutations in desmosomal proteins, suggesting that a common pathway is involved. Both TGF*β*3 and desmosomal proteins affect *β*-catenin signaling [1], and hence this could be an alternative pathway responsible for fibrosis. In this pathway cytoplasmic *β*-catenin is stabilized through Wnt signaling [49], after which *β*-catenin translocates to the nucleus, regulating gene transcription (cf. Figure 2).

For example, a homozygous loss-of-function mutation in *Jup* in a murine model of Naxos disease yielded symptoms that are also found in human ARVD/C [50]. In the *Jup* ^−/−^ mice, desmosomes were found to be absent, implying a role for plakoglobin and desmosomes in ARVD/C. Moreover, *Jup* ^+/−^ mice were found to develop ARVD/C at an age of 5 to 6 months [51]. These results demonstrate that a decrease in junctional plakoglobin is sufficient to cause ARVD/C over time. Notably, *Jup* was shown to activate Wnt signaling [52], thereby corroborating the notion of a role for Wnt signaling in ARVD/C. Similarly, silencing of *Dsp* mRNA, resulting in near total abolition of *Dsp* expression, was shown to suppress Wnt/*β*-catenin signaling [53]. Thus, reduced expression levels of *JUP* and *DSP*, encoding junctional plakoglobin and desmoplakin, respectively, might exert their role in ARVD/C through reduction Wnt/*β*-catenin signaling.

A role for Wnt signaling in fibrogenesis and adipogenesis has been suggested, as reviewed by Guo et al. [54]. Consistently, increased expression levels of genes involved in fibrotic and adipocyte development were observed in *Dsp*-diminished cells [53]. Further evidence comes from *in vitro* experiments showing that Wnt signaling inhibits adipogenesis [55]. Thus, Wnt/*β*-catenin suppression would result in increased levels of adipocyte development. In addition, activated Wnt/*β*-catenin signaling has been implicated in myogenesis as well as fibrogenesis [56, 57]. In combination, these findings suggest that alterations in the Wnt/*β*-catenin signaling pathway could be responsible for gradual fibrofatty infiltration.

TGF*β*-Smad signaling activates TGF*β*-activated kinase 1 (TAK1) and subsequently Nemo-like kinase (NLK) in the heart. In turn, NLK inhibits Wnt/*β*-catenin signaling by counteracting *β*-catenin-induced gene transcription [58]. Moreover, TGF*β* 1 and 3 have been shown to decrease E-cadherin expression via the Smad signaling pathway [59, 60]. This could alter cell-cell adhesion integrity, thereby ultimately resulting in suppressed *β*-catenin signaling, as was found in ARVD/C caused by mutations in *DSP* and *JUP*. Thus, TGF*β* inhibited *β*-catenin signaling could stimulate adipogenesis in cardiac tissue.

Alternatively, TGF*β*3 overexpression might be expected to have an activating role in the Wnt/*β*-catenin pathway. In human renal proximal tubular cells, TGFBR2 was shown to dissociate from adherens junctions upon TGF*β*1 activation [59]. TGF*β*3 is known to bind to the same receptor, possibly eliciting a similar process in the heart. Incubation with TGF*β*1 yielded increased levels of *β*-catenin, and dissociation of *β*-catenin from adherens junction complexes was observed [59]. In addition, the cytoplasmic *β*-catenin was stabilized by TGF*β* activity, thereby promoting Wnt/*β*-catenin signaling [54, 59]. This signaling pathway has been found to enhance fibrogenesis in cardiac tissue [61] or might even be necessary for TGF*β*-induced fibrosis [62].

At this point, the molecular mechanisms that might be involved in organ fibrogenesis are not properly defined [54]. There is still considerable uncertainty surrounding the direct effects of TGF*β*3 overexpression. Several pathways have been associated with TGF*β* signaling, sometimes with counteracting effects, as illustrated in Figure 2. The complexity is illustrated by Wnt/*β*-catenin signaling, as the effects of Wnt signaling are strongly dependent on the cell type and signaling context [61]. For this reason the effects of TGF*β* overexpression on Wnt signaling remain obscure. Further research is necessary to elucidate the complex interplay of TGF*β* signaling and Wnt signaling.

Finally, in both *Dsp* and *Jup* deficient mice, increased levels of apoptosis were observed [50, 53]. This might also be attributed to a decrease in Wnt/*β*-catenin signaling, which has been shown to be involved in regulation of apoptosis [63]. Hence, altered *β*-catenin signaling might be, at least in part, responsible for the increased levels of apoptosis observed in ARVD/C [64–66].

Therefore, one of the effects of TGF*β*3 might be exerted through its influence on cell-cell adhesion integrity, thereby altering *β*-catenin signaling in a variety of ways. Ultimately this can result in fibrofatty tissue development and apoptosis.

## 4. Apoptosis in ARVD/C Type 1

Myocardial damage is one of the hallmarks of ARVD/C. Signs of increased levels of apoptosis in myocardial tissue are frequently observed in patients suffering from ARVD/C [64–67]. Even though ARVD/C is rarely diagnosed in children below the age of puberty, increased levels of apoptosis have been reported in children over the age of 10 [67]. Yet not all patients suffering from ARVD/C show increased levels of apoptosis [64, 65], even though signs of fibrofatty infiltration are observed in the majority of patients [6]. This could be attributed to the episodic nature of apoptosis in ARVD/C [65]. It has been hypothesized that apoptosis of myocardial cells results in high levels of fibrofatty infiltration in the ventricular wall [64, 68], but progression is presumably episodic [65]. Mallat et al. [64] observed apoptosis primarily in myocardium that had not been affected by fibrofatty replacement. Hence, these observations suggest that apoptosis precedes replacement of myocardium with fibrofatty tissue, thus implying that apoptosis is not the result of the fibrofatty infiltration.

However, the origin of the increased level of apoptosis remains uncertain. Whether apoptosis in ARVD/C type 1 is a direct or secondary effect of the increased levels of TGF*β*3 remains to be established. TGF*β* signaling has been implicated in both cell growth and apoptosis, dependent upon the concentration of the cytokine and the signaling context. Myocardial cells of patients suffering from ARVD/C were shown to express and overexpress proapoptotic caspase 3 (CASP3) and Bax, respectively [64, 66]. Expression of these proteins resembles the mechanism thought to be involved in TGF*β*-induced apoptosis in other tissues, as reviewed by Schuster and Krieglstein [69]. Through binding of TGF*β* to a heterodimer of TGFBR1 and 2, Smad proteins are activated. Ultimately, CASP3 and subsequently the caspase cascade are activated through this pathway. Thus, TGF*β*3 overexpression might induce CASP3 expression, thereby lowering the threshold to apoptosis. Alternatively, altered Wnt/*β*-catenin signaling might also be involved, as discussed earlier.

Nevertheless, at this point it remains uncertain whether the aforementioned pathways are the main processes involved in myocardial apoptosis resulting from TGF*β*3 overexpression. Unraveling the source of myocardial degeneration is complicated by the subtle interplay of signaling pathways, and the multiple functions of TGF*β*3 [69]. Moreover, there has been no explanation for the episodic nature of apoptosis in ARVD/C. Also, the sensitivity of myocardial cells for altered levels of TGF*β*3 remains an enigma, as most other tissues seem to be unaffected [17]. More research to elucidate the underlying mechanisms would greatly improve our understanding of ARVD/C.

## 5. Inflammatory Injury in ARVD/C

Another contributor to the onset and progression of ARVD/C in general is inflammatory injury. Infiltration of inflammatory cells in necrotic or degenerative cardiac tissue has been reported in ARVD/C patients [23]. For example, Basso et al. [6] found patchy inflammatory infiltrates in 20 out of 30 ARVD/C hearts (67%). Furthermore, a correlation between the presence of inflammatory infiltrates and structural alterations of ventricular myocardium of ARVD/C samples has been reported by Campuzano et al. [70]. This study identified infiltration of mainly neutrophils and T-lymphocytes in the most severe cases of ARVD/C. Moreover, significant increases in inflammatory mediators are common findings in ARVD/C patients, which may reflect a disrupted balance between pro- and anti-inflammatory molecules [45]. However, the specific role of inflammation in the progression of macroscopic heart defects remains uncertain. Several mechanisms have been postulated, which are discussed below.

A frequent finding upon pathological examination of ARVD/C biopsies is myocarditis [6, 71]. It is acknowledged that myocarditis can be induced by a viral infection. Infiltration of inflammatory cells might be a response to pro-inflammatory cytokines induced by such a viral infection [14]. However, it seems unlikely that viral infection is the general cause for inflammation in ARVD/C. As one of the pathological changes in ARVD/C involves the degenerative loss of myocytes in ventricular myocardium [14, 71], an inflammatory response to myocardial tissue degradation seems a more appealing explanation.

Nevertheless, the role of viruses cannot entirely be excluded. Multiple studies report the presence of the enteroviral genome in the myocardium of ARVD/C patients with myocarditis, thereby supporting the possibility of its role in ARVD/C [71, 72]. Several of viral products have been demonstrated to induce apoptosis, for example, through activation of caspase enzymes as reviewed by Calabrese et al. [71]. Moreover, the immune response can further contribute to the loss of myocardial cells as a result of T-cell induced apoptosis, as reported by Huber [73]. Alternatively, factors excreted during the immune response can induce apoptosis. For example, elevated levels of tumor necrosis factor-*α* (TNF-*α*) and interleukin 1-*β* (IL1-*β*) converting enzyme result in production of nitric oxide synthase (iNOS) [71]. Ultimately, the stimulation of iNOS expression can result in apoptosis [74].

Genetic predispositions may amplify damage to myocytes caused by myocarditis, which in turn might quicken the progression of ARVD/C. However, it remains unclear whether inflammation and/or infection could be a primary cause of myocyte damage or a secondary response to degenerative loss of myocytes. Moreover, inflammation of cardiac tissue is not limited to the infiltration of inflammatory cells. Cardiac myocytes themselves produce inflammatory mediators and might thus contribute to immune-independent degeneration of cardiac tissue [45]. As discussed above, the biochemical context is a determining factor in both apoptosis and fibrosis. The inflammatory mediators and/or the excreted products from the immune system might create the appropriate conditions for the development of symptoms associated with ARVD/C.

Another possible mechanism by which inflammation plays a role in progression of ARVD/C might be the effect of inflammatory mediators on desmosomal proteins. As set out in the introduction, ARVD/C is predominantly caused by mutations in genes associated with cardiac desmosomes [1, 14]. Interestingly, Asimaki et al. [45] demonstrated that brief exposure to low concentrations of cytokines involved in myocarditis resulted in internalization of plakoglobin. This cytokine-induced redistribution of plakoglobin mimics the disruption of desmosomal structure. In this way, elevated inflammatory mediators may interfere with desmosomal function and integrity and hence the effects of TGF*β*3 overexpression, as in ARVD/C type 1, can be amplified. Such interference might contribute to severe myocardial injury, as observed in ARVD/C patients [70].

Taken all together, inflammation and/or infection may play a significant role in ARVD/C, possibly as a modulating and amplifying agent in disease progression. However, further research is required to elucidate the order in which certain pathogenic events occur; that is, whether inflammatory effects are a primary causal agent or a secondary response to already affected cardiac tissue. Moreover, a direct link to TGF*β*3 overexpression has not been established. Finally, inflammatory mediators in ARVD/C patients may be useful in risk stratification and possibly the development of anti-inflammatory therapies to ameliorate quality of life.

## 6. Concluding Remarks

The myocardial damage observed in ARVD/C is to a large extent due to the fibrofatty replacement and apoptosis of ventricular myocardium. TGF*β*3 overexpression, which is associated with ARVD/C type 1, might excessively activate cardiac fibroblasts through Smad signaling, resulting in fibrosis. Furthermore, TGF*β*3 overexpression may affect cell-cell adhesion integrity by dissociating *β*-catenin, thereby interfering with cell signaling pathways, resulting in fibrogenesis and apoptosis. Concerning the latter, overexpression of TGF*β*3 may stimulate CASP3 expression, resulting in a lowered threshold for apoptosis. Finally, inflammatory injury seems to play a significant role in progression of ARVD/C in general. The putative mechanisms seem to be interrelated, but at this point it is unknown whether these processes occur in concert, all induced by TGF*β*3 individually, or that some of the observations result indirectly from TGF*β*3 overexpression.

Although Beffagna et al. [26] demonstrated a clear cosegregation of ARVD/C type 1 with the mutation in the 5′ UTR region of the *TGF*β*3* gene, one may question whether TGF*β*3 overexpression is the actual molecular basis of ARVD/C type 1. One concern is that *in vivo *data on the (over) expression of TGF*β*3 in ARVD/C type 1 patients are lacking. Another concern involves the unsuccessful efforts to identify mutations in *TGF*β*3* in the two families of the original linkage analysis by Rampazzo et al. [25]. Of note, Rampazzo [75] recently reported that in one of these two families a large deletion encompassing the entire *PKP2* gene was identified. A further concern is the apparent cardioselective phenotype of ARVD/C type 1, despite the widespread tissue expression of *TGF*β*3*. The recently generated *Tgfb3* conditional knockout mouse [76] may prove helpful in addressing these concerns.

Revealing the molecular basis for ARVD/C remains to be a challenging task. In this review, an overview of putative mechanisms associated with onset and progression of ARVD/C, in particular type 1, has been provided, defining different directions to better understand the aforementioned molecular basis of the disease. However, research on ARVD/C type 1 is hindered by the small number of patients. This explains, at least in part, that the role of the aforementioned mechanisms has not been established. Even though the putative mechanisms are based on observations in ARVD/C patients as well as known interactions between TGF*β*3 and signaling pathways, their role is largely speculative. Therefore, further research is required to fully elucidate and verify the specific role TGF*β*3 induced signaling and the immune system in ARVD/C type 1 disease progression. A better understanding of ARVD/C can ultimately lead to better means for diagnosis and possibly it can offer possibilities for treatment of the disease.
